# Human genes: Time to follow the roads less traveled?

**DOI:** 10.1371/journal.pbio.3000034

**Published:** 2018-09-26

**Authors:** Ian Dunham

**Affiliations:** Open Targets and European Molecular Biology Laboratory, European Bioinformatics Institute, Wellcome Genome Campus, Hinxton, Cambridge, United Kingdom

## Abstract

Determining the functions of human genes is a key objective for understanding disease and enabling development of new therapeutic approaches. A number of recent studies have shown that the amount of attention the research community gives to each of the more than 20,000 human genes is dramatically skewed toward specific, well-known genes. In this issue, Stoeger and colleagues uncover the factors that explain this bias and offer a way ahead to move more genes into the research limelight.

## Abstract 2

Some genes get all the luck. This Primer explores a new analysis as to why the amount of research attention given to each of the more than 20,000 human genes is dramatically skewed towards specific, well known genes, and asks whether we need to take steps to change it.

There is a specific type of observational bias in which we only look for something for which the search is easiest. Known as the street light effect, it has been recognized in popular anecdotes since at least the 1920s [1], as well as being widely illustrated in cartoon form. In these days of genome sequences and high-throughput biology, surely this couldn’t be happening when we study human genes, could it? Incredibly, a new study by Stoeger and colleagues [2] published here suggests that it is.

With the completion of the human genome sequence [3], efforts to itemize human genes [4] have settled on a set of around 20,000 protein-coding genes [5]. Estimates of the number of genes that do not code for proteins, particularly long noncoding RNAs, are more fluid, but the best estimate from a highly curated annotation set is just under 16,000 [5]. So at least for the protein-coding genes, we have defined a parts list from which to study function. In the pregenomics era, determining the sequence of a single gene could be the topic of a whole PhD thesis or the focus of a single lab, and there is no doubt that detailed hypothesis-driven studies of single genes continue. However, new high-throughput technologies to assay transcript and protein expression, the effects of diverse gene knockdowns and knockouts, or the associations of natural human population variation with disease have opened up the possibility of unbiased assignment of function to genes. So all things being equal, we should expect to see comprehensive functional annotation distributed across the full range of human genes. Of course, the reality is not like that at all.

Several publications over the last 15 years [6–11] have observed that the patterns of publications on human genes are highly skewed. Certain genes, such as TP53, become fashionable and then tend to dominate the published literature year after year. This imbalance could be for a number of reasons, including the intrinsic properties of the genes, technological or reagent limitations, medical relevance, or complex social and economic factors affecting research priorities. Stoeger and colleagues set out to unpick these relationships by assembling a set of 430 computed or experimentally determined gene properties and then constructing models that predict the number of publications and the date of first publication for each of the approximately 13,000 human genes for which they had full data. Using a machine-learning technique (gradient boosting regressions with out-of-sample Monte Carlo cross-validation), they could predict the number of publications per gene with reasonable accuracy (Spearman rank correlation: 0.64). Just 15 of the gene features dominated the model’s accuracy, representing aspects of RNA and protein abundance, transcript and gene length, protein sequence factors including the presence of a signal sequence, and the sensitivity of the gene to natural or gene-edited mutations. Thus, it seems that the overall research activity on each human gene as judged by the total number of publications is substantially influenced by properties of genes that affect their tractability by multiple experimental methods.

Remarkably, the authors also show that this skewing of interest toward specific genes is consistent over time and that genes that were initially reported on early tend to continue to accrue enhanced attention through more publications. For instance, the 16% of human genes with publications before 1991 generate 49% of publications in 2015. As the authors and others put it, the rich get richer. What is more, the same factors used to predict the number of publications on a human gene can, together with information on the initial publication date on its orthologues in model organism, be used in a model that accurately predicts the first year of publication on the human gene. These same models can also predict the allocation of National Institutes of Health (NIH) funding to grants for human gene research and the existence of approved and preclinical drugs with the gene as the drug target. Taken as a whole, these results suggest that much of the direction for basic and applied research for human genes and disease is influenced by favorable characteristics of the genes and the availability of model organism data, i.e., we are looking under the street light.

One could argue that we are concentrating on the most interesting genes, which are most likely to be involved in human disease. Indeed, Stoeger and colleagues test this and show that, to some extent, research is focusing on the genes most likely to be sensitive to loss of function mutations or to be identified in genome-wide association studies (GWAS) of human disease. However, after taking this into account, there is still a shockingly disproportionate concentration on the genes that have already been studied most.

The advent of genome sequences and unbiased approaches to data generation has given us the ability to survey phenotypic effects across all genes. In certain areas, such as GWAS, this has succeeded spectacularly [12], but we are all aware of the tendency to cling to what we know when examining large amounts of data. The slide of an impenetrable network analysis hairball with a few familiar named nodes picked out to provide validation must be familiar to many. Despite these advances, Stoeger and colleagues show that the patterns of focusing on the already well studied has continued over the last decade. However, there is some hope because single gene studies that refer to unbiased studies across many genes tend to focus on more understudied genes than would otherwise be expected. Helpfully, the authors also try to assist us to escape our attention biases with supplementary information that can guide us toward neglected genes that already have suitable data that might aid their study.

None of this would matter if we knew that we were definitely studying the “right” genes, but GWAS and studies of rare human disease continue to throw out associations to previously neglected genes. There is also a pressing need to identify new drugs and, by implication, new drug targets for a range of unmet need in human disease, while drug targets with genetic validation are more likely to seed a successful drug discovery program [13]. It doesn’t seem too fantastic to think that there are potentially rich pickings of drug targets with genetic validation lying beyond the street light. The Illuminating the Druggable Genome program has gone some way down this route by focusing on neglected proteins from the approximately 3,000 members of protein families of which other members have been successful drug targets (kinases, G-protein-coupled receptors [GPCRs], and ion channels) [14]. At Open Targets, we have gone further by bringing together in a single platform [15], https://www.targetvalidation.org, public data across all human genes, including genetics pertinent to drug target identification, as well as performing genome-wide experiments that can identify the causal effect of genes on relevant phenotypes [16]. We anticipate that these unbiased approaches can shift the focus of drug target identification and prioritization toward genes with higher chances of underpinning successful drug discovery programs beyond the usual suspects.

Of course, we can’t give up on the study of well-studied genes because there is still so much about their mechanisms that remains to be learned. Many years ago, my graduate study was in a unit that studied the complement genes, and I contributed in a small way to unpicking some of the complex structural variation of the complement C4 genes within the human MHC [17]. A great deal was known even then about the role of C4 in the complement pathway of the innate immune system, and while there were some indications of potential roles in autoimmune disease, the genetics, biochemistry, and function of C4 seemed well established [18]. There was no inkling of what remained to be discovered. Skipping ahead nearly 30 years, I was astonished when McCarroll and colleagues [19] were able to show a new role for C4 gene variation in schizophrenia. The strongest GWAS signal for schizophrenia lies in the MHC region, which is notoriously difficult to study because of the strong linkage disequilibrium across the complex. However, by systematically unpicking the configurations of the C4 genes across patients and controls, McCarroll and colleagues were able to show the causal role of complex variation in the C4 genes, implicating a role for increased complement activity in schizophrenia. So surprising avenues await even down the more traveled roads, and the lessons learned from years of research on specific genes in one area can enlighten our understanding when placed in a new context.

Nevertheless, Stoeger and colleagues provide a timely reminder that the choices we make in our research on human genes are limiting our understanding of the full complement of the human genome. Surely, many opportunities are being missed by this omission. It’s time to follow some of the roads less traveled.

## Abbreviations

GWAS: genome-wide association studies
GPCR: G-protein-coupled receptor
NIH: National Institutes of Health
